# 4‐octyl itaconate improves the viability of D66H cells by regulating the KEAP1‐NRF2‐GCLC/HO‐1 pathway

**DOI:** 10.1111/jcmm.17708

**Published:** 2023-03-13

**Authors:** Yanrui Chen, Zhenying Wang, Yali Song, Nan Chen, Jing Guo, Wenmin Liu, Keying Guo, Xia Ling, Li Zhang

**Affiliations:** ^1^ Department of Dermatology Shandong Provincial Hospital Affiliated to Shandong First Medical University Jinan China; ^2^ Department of Dermatology, Shandong Provincial Hospital, Cheeloo College of Medicine Shandong University Jinan China

**Keywords:** 4‐octyl itaconate, apoptosis, D66H, NRF2, oxidative stress

## Abstract

As a novel nuclear factor E2‐related factor 2 (NRF2) activator, the itaconate has shown significant therapeutic potential for oxidative stress diseases. However, its role in Vohwinkel syndrome in relation to the *gap junction protein beta 2* (*GJB2*) mutation is still unclear. This study aimed at investigating the effect of 4‐octyl itaconate (OI) on HaCaT and D66H cells and clarify its potential mechanism in vitro. The optimal concentration and treatment time of OI on HaCaT cells and D66H cells were determined by CCK‐8 and LDH experiments. The effect of OI on cell proliferation was detected by EdU staining and FACS analysis of PI, while the apoptosis was evaluated by TUNEL staining and FACS analysis of Annexin V. The ROS staining was performed, and the levels of SOD, MDA, GSH and GSH/GSSG were detected to evaluate the effect of OI on oxidative damage induced by D66H‐type mutation. CO‐IP, Western blot, immunofluorescence and qPCR analyses were employed to detect the activation of KEAP1‐NRF2‐GCLC/HO‐1 pathway by OI. Finally, sh‐NRF2 was used to confirm the activation of this pathway by OI. Results showed that OI could improve the cell viability decreased by *GJB2* gene mutation by regulating the balance between cell growth and apoptosis induced by oxidative damage. Furthermore, this alleviation process was regulated by the KEAP1‐NRF2‐HO‐1/GCLC pathway. In conclusion, OI could improve the viability of HaCaT and D66H cells via regulating the KEAP1‐NRF2‐GCLC/HO‐1 pathway, which provided a wide spectrum of potential targets for effective therapeutic treatments of Vohwinkel syndrome in the clinic.

## 1 INTRODUCTION

As a rare and refractory hereditary skin disease belonging to the familial and autosomal dominant dermatosis, the Vohwinkel syndrome (VS) is characterized by diffuse hyperkeratosis in the palms of hands and soles of feet, which would develop to auto‐amputation if not treated adequately.^1^, ^2^ This disease is usually accompanied by many other medical disorders, for example sensorineural deafness, ichthyosis, vitiligo and mental retardation, severely affecting the patients' physical and mental health.^2^ However, the effective therapeutic treatment of VS is still lacking in the clinical settings.^3^

Recent studies have shown that there are two types of palmoplantar keratoderma mutilating. One is an ichthyosis‐related variant caused by the insertional mutations in the loricrin gene.^4^, ^5^, ^6^ The other is a deafness‐related variant associated with Connexin‐26 (Cx‐26), which is the gap connection protein responsible for signal transmission and material exchange between adjacent cells and encoded by the *gap junction protein beta 2* (*GJB2*) gene.^7^, ^8^, ^9^, ^10^, ^11^ To date, a group of five *GJB2* gene mutation sites have been reported to cause the classical VS, include p.Asp66 His (D66H), p.Gly130 Val (G130V), p.Gly59 Ser (G59S), p.Tyr65 His (Y65H) and p.Arg75 Gln (A75H), with the p.Asp66 His (D66H) as the first mutation type discovered and defined.^12^, ^13^, ^14^, ^15^ The human immortal keratinocyte line (HaCaT) cells are the well‐established human immortalized keratinocyte in vitro model based on D66H‐type mutation with a high survival rate and are commonly used for genetic and molecular investigations of skin diseases.^9^

Studies have shown that the deletion or mutation in *GJB2*, which is also an oxidative stress‐related gene, would activate cellular oxidative stress mainly by downregulating the nuclear factor E2‐related factor 2 (NRF2) pathway.^16^ As a transcription factor, the NRF2 plays a crucial role in the inflammatory responses and oxidative stress.^17^, ^18^, ^19^ Under normal physiological conditions, the NRF2 is inactive by binding to Kelch‐like ECH‐associated protein l (KEAP1) in the cytoplasm.^20^ With the presence of either endogenous or exogenous stimuli, the NRF2 is dissociated from KEAP1 and transferred into the nucleus to regulate the target genes, such as heme oxygenase 1 (*HO‐1*), glutamate‐cysteine ligase catalytic subunit (*GCLC*) and NAD(P)H quinone dehydrogenase 1 (*NQO1*), ultimately regulating the production of glutathione (GSH).^20^, ^21^ These findings imply that NRF2 is an indispensable signalling factor that regulates oxidative stress to affect cell fate with the *GJB2* dysfunctions. Previous studies have shown that deletion or mutation of the *GJB2* gene cause the oxidative stress to accelerate apoptosis in skin cells.^16^, ^22^, ^23^ Therefore, the NRF2 could be a promising target in the treatment of hereditary skin disease caused by the *GJB2* dysfunction.

As an intermediate metabolite of the tricarboxylic acid (TCA) cycle, the itaconate has been revealed with antioxidant effects by attenuating the intracellular succinate dehydrogenase (SDH) activity.^20^ Recent studies have shown that the itaconate could promote the alkylation of KEAP1, thereby promoting the dissociation of NRF2 from KEAP1, leading to the activation of NRF2.^20^ Itaconate belongs to the α, β unsaturated carboxylic acids with electrophilicity, which enables itaconate to interact with proteins containing sulphur groups at the cellular level, a process known as an electrophilic stress response (ESR).^24^ Due to the ESR, the 4‐Octyl itaconate (OI) contains an ester group, which makes the OI penetrate the cells at a high transformation rate without the assistance of transporting protein.^20^ Because itaconate could activate NRF2, OI has also been shown to play an antioxidant role to improve the cell survival.^20^ For example, OI could improve the LPS‐induced chondrocyte inflammation and attenuate the H2O2‐induced neuronal reactive oxygen species (ROS) generation and lipid oxidation.^25^, ^26^ Furthermore, OI could also improve the prognosis in prebrain/liver ischemia–reperfusion injury and inhibit UVB‐induced oxidative stress in melanocytes and keratinocytes.^27^, ^28^, ^29^ These studies have revealed the sound antioxidant effects and protection of OI. However, the role of OI in the destructive keratoderma has not been studied; in particular, its involvement in the NRF2 signalling pathway is still unknown.

We hypothesize that OI can prevent VS induced by the D66H‐type mutation and may mediate the protection via activating the KEAP1‐NRF2‐GCLC/HO‐1 signalling pathway based on study findings of OI in other disease models. This study aimed at investigating the effect and mechanism of OI on D66H‐type mutation‐induced VS in vitro to explore the potentially effective diagnosis and therapeutic treatment of VS in the clinical settings.

## 2 METHODS

### 2.1 Cell culture and treatments

The HaCaT cells were purchased from Sunncell (SNL‐163, Wuhan, China). The *GJB2* mutant cell line (D66H) was synthesized by Genechem (Shanghai, China) through transfection into HaCaT cells. All stable cell lines were thoroughly screened by 1 μM Geneticin sulfate (G‐418, HY‐17561, MCE). Both the HaCaT and D66H cell lines were cultured in DMEM (11,965,092, Gibco) containing 10% (v/v) foetal bovine serum (10,091,148, Gibco) in 5% CO_2_ at 37°C.

### 2.2 CCK‐8 assay

In the 96‐well culture plates, 1.0 × 10^4^ HaCaT or D66H cells were seeded per well and cultured for 12 h to cover 75% of the area of the bottom of the well. The cells were treated with OI for 24 h. Then, the cells were grown in 100 μL of complete medium for 1 h at 37°C and supplemented with 10 μL of CCK‐8 working fluid (CKO4, DOJINDO Laboratory). The cytotoxicity rate was determined by measuring the absorption rate of the sample at 450 nm using a microplate reader (Multiskan GO, Thermo Scientific).

### 2.3 Lactate dehydrogenase (LDH) assay

The absorbance of different groups of cells at 490 nm was measured by the LDH Cytotoxicity Assay Kit (C0016, Beyotime Biotechnology) with the relative content of intracellular lactate dehydrogenase (LDH) calculated. The data were normalized based on the HaCaT Control group (HC).

### 2.4 EdU staining

Both the cells in the supernatant and adherent state were collected, fixed with 1% paraformaldehyde solution (PFA), blocked with 70% ethanol at −20°C for 24 h and stained with 0.5 mL PI/RNase Staining Buffer (550,825, BD) at room temperature for 15 min. The cells were detected by flow cytometry (FACS Celesta, BD). Modfit LT software (Version: 5.0, https://mybiosoftware.co) was used to examine the samples from each cycle to determine the distribution of cells.

### 2.5 Cell proliferation assay

In the 24‐well culture plates, the cells covering 75% of the entire bottom of each well were added with OI for 24 h. Both the cells in the supernatant and adherent state were collected, fixed with 1% paraformaldehyde solution (PFA), blocked with 70% ethanol at −20°C for 24 h and stained with 0.5 mL PI/RNase Staining Buffer (550,825, BD) at room temperature for 15 min. The cells were detected by flow cytometry (FACS Celesta, BD). Modfit LT software (Version: 5.0, https://mybiosoftware.com) was used to examine the samples from each cycle to determine the distribution of cells.

### 2.6 TUNEL staining

The One Step TUNEL Apoptosis Assay Kit (MA0224, Meilunbio) was used to measure the percentage of dead cells in HaCaT or D66H cultures by following the manufacturer's instructions. The cells were permeabilized with proteinase K at a concentration of 20 g/mL in order to enhance the efficacy of the staining, which was then followed by the application of DAPI and TUNEL staining. The confocal fluorescence microscope (TCS SP8, Leica) was used to capture the images, and ImageJ was used to determine the fluorescence counts on the data (Version: 2.0.0‐rc‐69/1.52p, http://imagej.net/Contributors).

### 2.7 Cell apoptosis assay

Trypsin without EDTA was used to suspend the cells with the cells floating in the supernatant collected at the same time. Each sample undergo a reaction with 5 μL of PE Annexin V and 7‐ADD for 15 min using the PE Annexin V Apoptosis Detection Kit I (559,763, BD) by following the manufacturer's guidelines. The excitation voltage was modified to the appropriate levels based on the size and shape of the cells. Flow cytometry was used to count the number of dead cells (FACS Celesta, BD).

### 2.8 Reactive oxygen species (ROS) assay

The cells were stained for 30 min with DCFH‐DA dye in a 5% CO2 incubator at 37°C using the ROS Assay Kit (Photo‐oxidation Resistant DCFH‐DA, R253, DOJINDO) by following the manufacturer's instructions. The nucleus was stained with Hoechst 33,342 for cell localization. The images were captured using a confocal fluorescence microscope (TCS SP8, Leica), and the fluorescence intensity was calculated using ImageJ (Version: 2.0.0‐rc‐69/1.52p, http://imagej.net/Contributors).

### 2.9 Intracellular superoxide dismutase (SOD) assay

An Enhanced BCA Protein Assay Kit was used to measure the protein content of cell lysates (P0009, Beyotime Biotechnology). The absorbance of each sample was measured at 520 nm, and the enzymatic activity of SOD was calculated according to the protocol provided by the Total Superoxide Dismutase Assay Kit with WST‐8 (S0101S, Beyotime Biotechnology).

### 2.10 Lipid peroxidation assay

The Enhanced BCA Protein Assay Kit was first used to measure the protein content of cell lysates (P0009, Beyotime Biotechnology). After the treatment with Malondialdehyde (MDA) working solution, the sample was heated in a 100°C water bath for 10 min by following the instructions of the Lipid Peroxidation MDA Assay Kit (S0131S, Beyotime Biotechnology). The absorbance of each sample was then determined at 532 nm. Standard curve analysis was used to determine the MDA levels in the samples.

### 2.11 GSH/GSSG measurements

In the 96‐well culture plates, 1.0 × 10^4^ HaCaT or D66H cells were seeded per well and cultured for 12 h to cover 75% of the bottom area of each well. Each sample was treated with 50 ul of GSH working solution for 5 min at room temperature. The GSH content and the GSH/GSSG ratio were determined based on the absorbance at 412 nm and a GSH and GSSG assay kit (S0053, Beyotime Biotechnology).

### 2.12 Co‐immunoprecipitation (CO‐IP)

A total of 600 μg or more protein lysates per sample were collected. The lysates were precleared with control agarose resin in the Pierce Co‐Immunoprecipitation (Co‐IP) Kit (26,149, Thermo SCIENTIFIC). Then, the Keap1‐binding protein was captured by either anti‐Keap1 antibody (4678, CST, 1:50) or protein IgG beads, and the KEAP1‐NRF2 immune complexes were detected by Western blot analysis.

### 2.13 Western blot analysis

The HaCaT or D66H cells were cultured at a density of 2.0 × 10^6^ cells per dish in 100 mm Cell Culture Dishes. The cellular proteins were extracted using RIPA buffer (R0020, Solarbio) containing 1% (v/v) PMSF (P0100, Solarbio). An Enhanced BCA Protein Assay Kit was used to measure the protein content (P0009, Beyotime Biotechnology). The instructions of the Nuclear Protein Extraction Kit (R0050, Solarbio) were strictly followed for the extraction of cytoplasmic and nuclear proteins. The protein sample as an equal amount was denatured and separated by SDS‐PAGE electrophoresis and transfected into 0.2 μm PVDF (ISEQ00010, Merck Millipore). The primary and secondary antibody working fluids were prepared according to the instructions of the antibody manual and incubated at 4°C overnight and at room temperature for 2 h, respectively. The PVDF was exposed after staining with the ECL Kit (WBKLS0500, Merck Millipore). ImageJ software was used to quantify the grey value of the stripe (Version:2.0.0‐rc‐69/1.52p, https://imagej.net/Contributors). The primary and secondary antibodies included mouse anti‐β‐actin (TA‐09, ZSGB‐BIO, 1:1000), rabbit anti‐lamin B1 (ab16048, Abcam, 1:1000), rabbit anti‐NRF2 (ab62352, Abcam, 1:2000), rabbit anti‐GCLC (ab207777, Abcam, 1:1000), rabbit anti‐heme oxygenase 1 (ab52947, Abcam, 1:1000), rabbit anti‐KEAP1 (4678, CST, 1:1000), horseradish enzyme‐labelled goat anti‐rabbit IgG (ZB‐2301, ZSGB‐BIO) and horseradish enzyme‐labelled goat anti‐mouse IgG (ZB‐2305, ZSGB‐BIO).

### 2.14 Immunofluorescence staining

After the treatment with OI, the HaCaT and D66H cells were fixed by 4% PFA (P1110, Solarbio) for 30 min. Prior to the blocking with 5% BSA or serum homologous to the secondary antibody, the 0.3% Triton X‐100 (T8200, Solarbio) was permeabilized for 30 min. Working fluids for the primary and secondary antibodies were prepared and incubated at 4°C overnight and at room temperature for 2 h, respectively, by following the antibody instructions. In particular, the secondary fluorescent antibody and DAPI (C0065, Solarbio) staining were incubated in the dark. Antifade Mountant (P36930, Invitrogen) was used to preserve the cells before they were viewed and photographed using an inverted laser scanning confocal fluorescence microscope (Leica, TCS SP8, Leica). The primary fluorescent antibodies were the same as those used in the Western blot analysis. The secondary fluorescent antibodies included CoraLite488‐goat Anti‐rabbit IgG (H + L) (SA00013‐2, proteintech) and CoraLite594‐goat Anti‐rabbit IgG (H + L) (SA00013‐4, proteintech).

### 2.15 Quantitative Real‐time PCR (qPCR)

The total cellular RNA was extracted using TRIzol (15,596,018, Ambion). By following the manufacturer's instructions, cDNA was synthesized with an Evo M‐MLV RT Mix Kit with gDNA Clean for qPCR (AG11728, ACCURATE BIOLOGY). The qPCR was performed with SYBR Green Premix Pro TagHS qPCR Kit (AG11701, ACCURATE BIOLOGY) and fluorescence quantitative PCR instrument (480, LightCycler). The fold changes of different groups of samples were calculated by the ΔΔCT method, with the data normalized to the HC group. The primers used in these experiments were listed in Table 1.

**TABLE 1:** Primer sequences used for qPCR. ‘F’ and ‘R’ indicate forward and reverse primers, respectively.

Genes	Primer sequences
Human *GAPDH*	F: 5'–GCACCGTCAAGGCTGAGAAC–3'
	R: 5'–TGGTGAAGACGCCAGTGGA–3'
Human *NFE2L2*	F: 5'–ATCCATTCCTGAGTTACAGTGTCTT–3'
	R: 5'–TGTCAGTTTGGCTTCTGGACT–3'
Human GCLC	F: 5'–TCCAGGTGACATTCCAAGCC–3'
	R: 5'–GCAGCACTCAAAGCCATAACA–3'
Human *HO‐1*	F: 5'–TGCTGACCCATGACACCAAG–3'
	R: 5'–GGGCAGAATCTTGCACTTTGTT–3'

### 2.16 NRF2 shRNA transfection of HaCaT and D66H cells

Three ‘NRF2‐shRNA’ lentiviral plasmids (i.e. si‐69, si‐70 and si‐71) were developed and generated based on the human NFE2L2 gene and a negative nonsense control ‘NC’ (Genomeditech, Shanghai, China). The cells were grown in a complete culture medium containing the shRNA lentivirus for 48 h. Puromycin of 0.1, 0.5, 1.0 and 2.0 g/mL was used to screen stable cells. Both Western blot and qPCR analyses were performed to verify the reduction of NRF2.

### 2.17 Statistical analysis

Each experiment was repeated with three or more biological replicates. Data were presented as mean ± standard deviation (SD) with the one‐way analysis of variance (anova) and independent *t*‐test performed to determine the significant difference based on *p* < 0.05, using the Graph Pad Prism (Version: 9.4.0 (453), https://www.graphpad‐prism.cn/).

## 3 RESULTS

### 3.1 OI alleviates the cell viability decline and cell proliferation arrest caused by *GJB2* gene mutation

**FIGURE 1:**
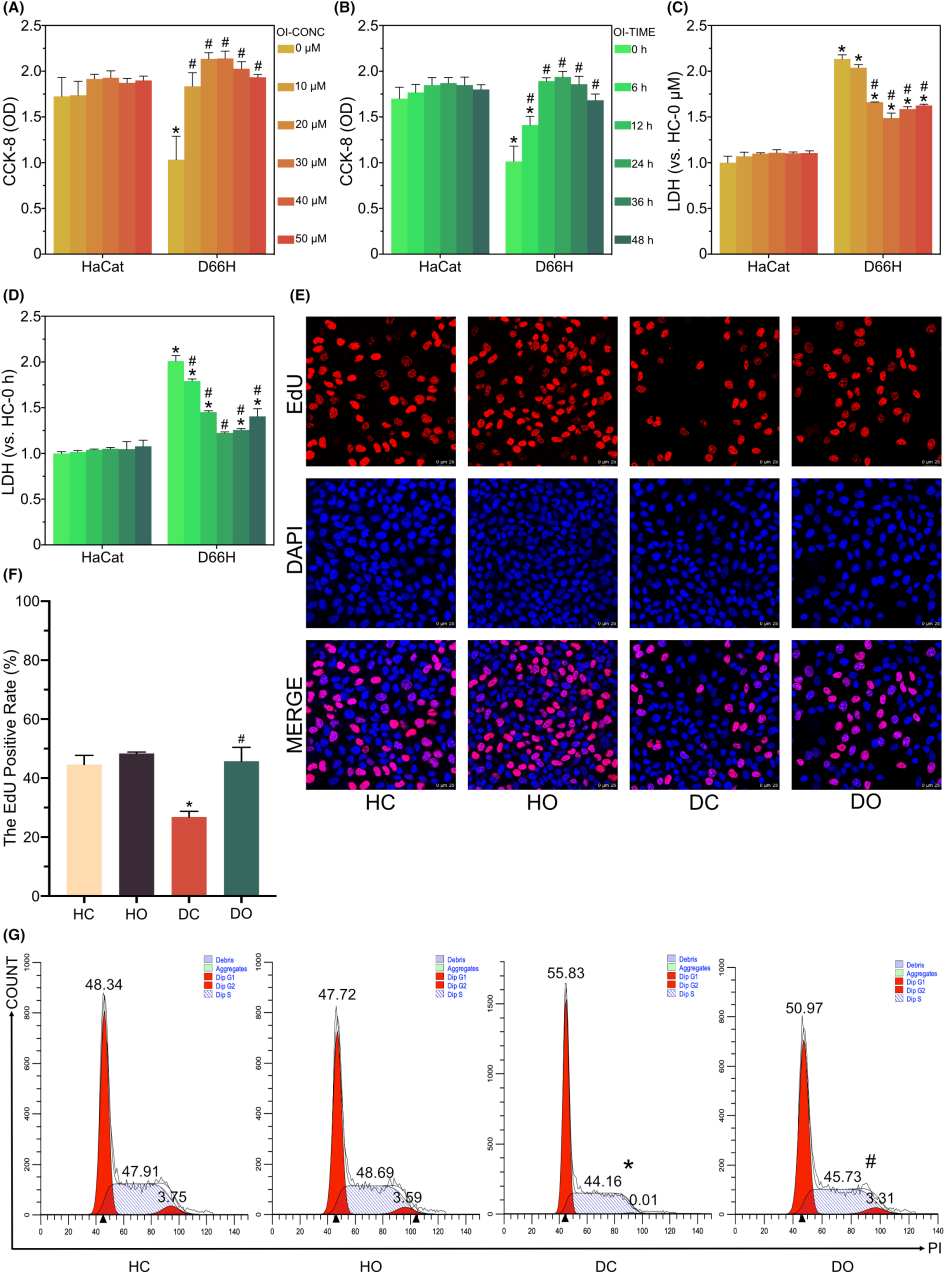
Alleviation effect of 4‐octyl itaconate (OI) on cell viability decline and cell proliferation arrest caused by *GJB2* gene mutation. The effect of different OI concentrations (i.e., 0, 10, 20, 30, 40, and 50 μM) for 12 h on the viability of HaCaT cells and D66H mutant cells (A). The effect of different OI treatment times (i.e., 0, 6, 12, 24, 36, and 48 h) at concentration of 30 μM on the viability of HaCaT cells and D66H mutant cells (B). The effect of different OI concentrations (i.e. 0, 10, 20, 30, 40 and 50 μM) for 12 h on the LDH levels of HaCaT cells and D66H mutant cells (C). The effect of different OI concentrations (i.e. 0, 10, 20, 30, 40 and 50 μM) for 12 h on the LDH levels of HaCaT cells and D66H mutant cells (D). The EdU staining and analysis of proliferating cells (E, F). The FACS analysis of cell cycle and proportion of each phase of the cell cycle (G). All experiments are based on HaCaT control group (HC), HaCaT OI treatment (30 μM and 24 h) group (HO), D66H control group (DC) and D66H OI treatment (30 μM and 24 h) group (DO). The significant difference is determined based on *p* < 0.05 (*) compared with HC and *p* < 0.05 (#) compared with DC.

Each group of cells were treated with OI at a variety of concentrations (0, 10, 20, 30, 40 and 50 μM) and treatment times (0, 6, 12, 24, 36 and 48 h) to determine the optimal concentration and treatment time of OI. The results of CCK‐8 assay showed that compared with the control group (DC), the absorbance was significantly decreased in D66H cells with no treatment of OI. In contrast, the release of LDH was evidently increased, while no cytotoxicity was observed with the OI treatment alone of the appropriate concentration and treatment time (Figure 1A–D). Additionally, the OI treatment enhanced the survival of D66H‐type mutant cells in a dose‐ and time‐dependent manner (Figure 1A–D). Notably, OI showed a strong protective effect at 30 μM and 24 h. Therefore, the concentration of 30 μM and treatment of 24 h were chosen for use in the following experiments.

Next, the EdU staining experiments were performed to further explore the molecular mechanism regulating the viability of HaCaT and D66H cells. Immunofluorescence analysis revealed that the proportion of D66H‐type mutant cells showing proliferation was much lower than that of wild‐type HaCaT cells. Conversely, the OI treatment resulted in an evident increase in the proportion of proliferating cells (Figure 1E,F). These results implied that OI stimulated cell growth in D66H cells. The results of flow cytometry showed that the percentage of D66H cells in the G0/G1 phase was significantly greater than that in the wild‐type HaCaT cells, while the percentage of cells in the G2/M phase was increased in the OI‐treated group (Figure 1G). These results indicated that OI ameliorated the G1 phase arrest caused by the D66H‐type mutation.

### 3.2 OI inhibits apoptosis induced by *GJB2* gene mutation

**FIGURE 2:**
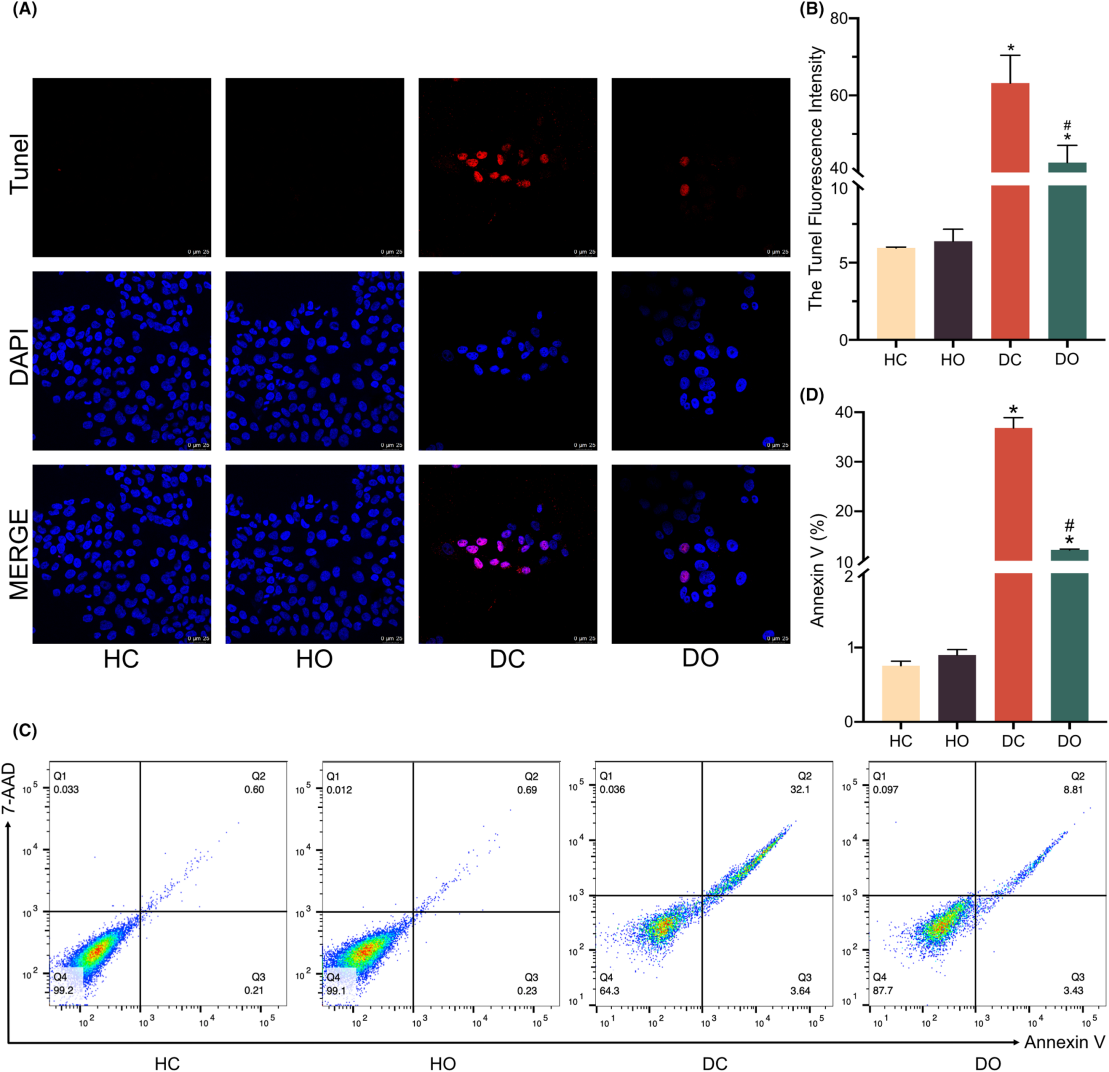
Inhibitory effect of 4‐octyl itaconate (OI) on apoptosis induced by *GJB2* gene mutation. The TUNEL staining and fluorescence intensity analyses (A, B). The FACS analysis of apoptosis and the ratio of Annexin V (C, D). All experiments are based on HaCaT control group (HC), HaCaT OI treatment (30 μM and 24 h) group (HO), D66H control group (DC) and D66H OI treatment (30 μM and 24 h) group (DO). The significant difference is determined based on *p* < 0.05 (*) compared with HC and *p* < 0.05 (#) compared with DC.

The results of the TUNEL fluorescence assay and the annexin V‐7AAD FACS assay demonstrated that the proportions of TUNEL‐positive and Annexin V‐positive cells in the DC group were significantly higher than that in the HC group (Figure 2A–D **)**, providing evidence to support that the D66H mutation caused apoptosis in the HaCaT cells. With the treatment of OI, a significant reduction was observed in the proportions of both TUNEL‐positive and Annexin V‐positive cells in D66H cells of the HC group (Figure 2A–D). Apoptosis was not observed in HaCaT cells treated with OI alone. These findings suggested that OI appeared to protect cells against the apoptosis caused by the D66H mutation.

### 3.3 OI prevents oxidative damage implemented by *GJB2* gene mutation

**FIGURE 3:**
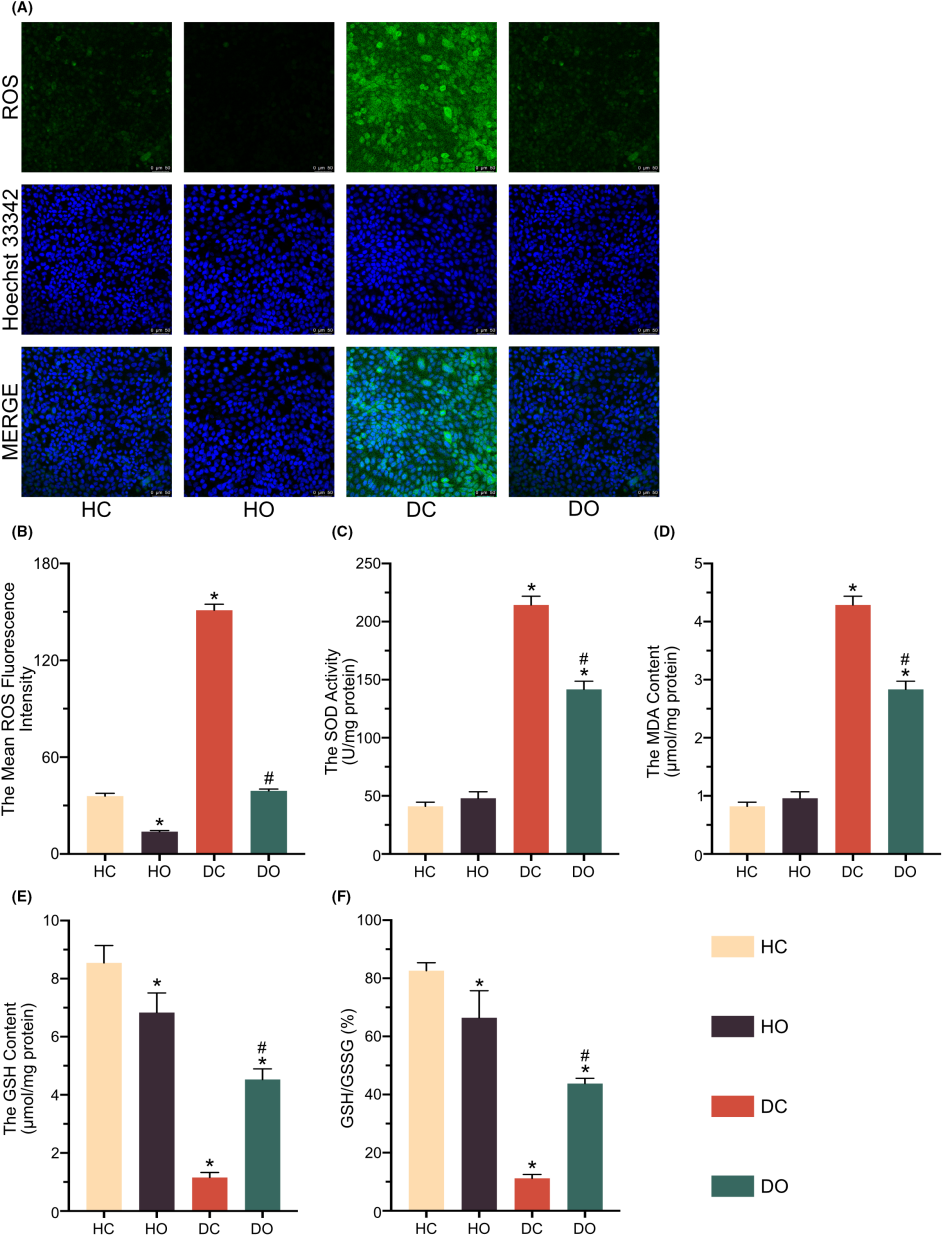
4‐octyl itaconate (OI) prevents oxidative damage implemented by *GJB2* mutation. The ROS staining and mean fluorescence intensity analysis (A, B). The Superoxide Dismutase (SOD) activity (C). The Lipid Peroxidation MDA Content (D). The GSH content and the ratio of GSH/GSSG (E, F). All experiments are based on HaCaT control group (HC), HaCaT OI treatment (30 μM and 24 h) group (HO), D66H control group (DC) and D66H OI treatment (30 μM and 24 h) group (DO). The significant difference is determined based on *p* < 0.05 (*) compared with HC and *p* < 0.05 (#) compared with DC.

The fluctuating levels of oxidative stress were detected in both HaCaT and D66H cells either with or without the presence of OI. The D66H‐type mutation induced excessive cell ROS production (Figure 3A,B). The D66H cells exhibited the increased levels of both SOD and lipid peroxidation (Figure 3C,D). Simultaneously, the relative content of GSH and the GSH/GSSG ratio was reduced by the D66H mutation (Figure 3E,F). The effect of OI on the oxidative stress was observed. The fluorescence intensity of ROS was significantly decreased following the OI treatment, so were the contents of SOD and the lipid peroxide activities, whereas the level of the antioxidant GSH was dramatically increased (Figure 3A–F). In addition, the basal oxidation level was not changed in the HaCaT cells treated by the OI (Figure 3A–F).

### 3.4 OI activates the KEAP1‐NRF2‐HO‐1/GCLC signalling pathway in HaCaT cells

**FIGURE 4:**
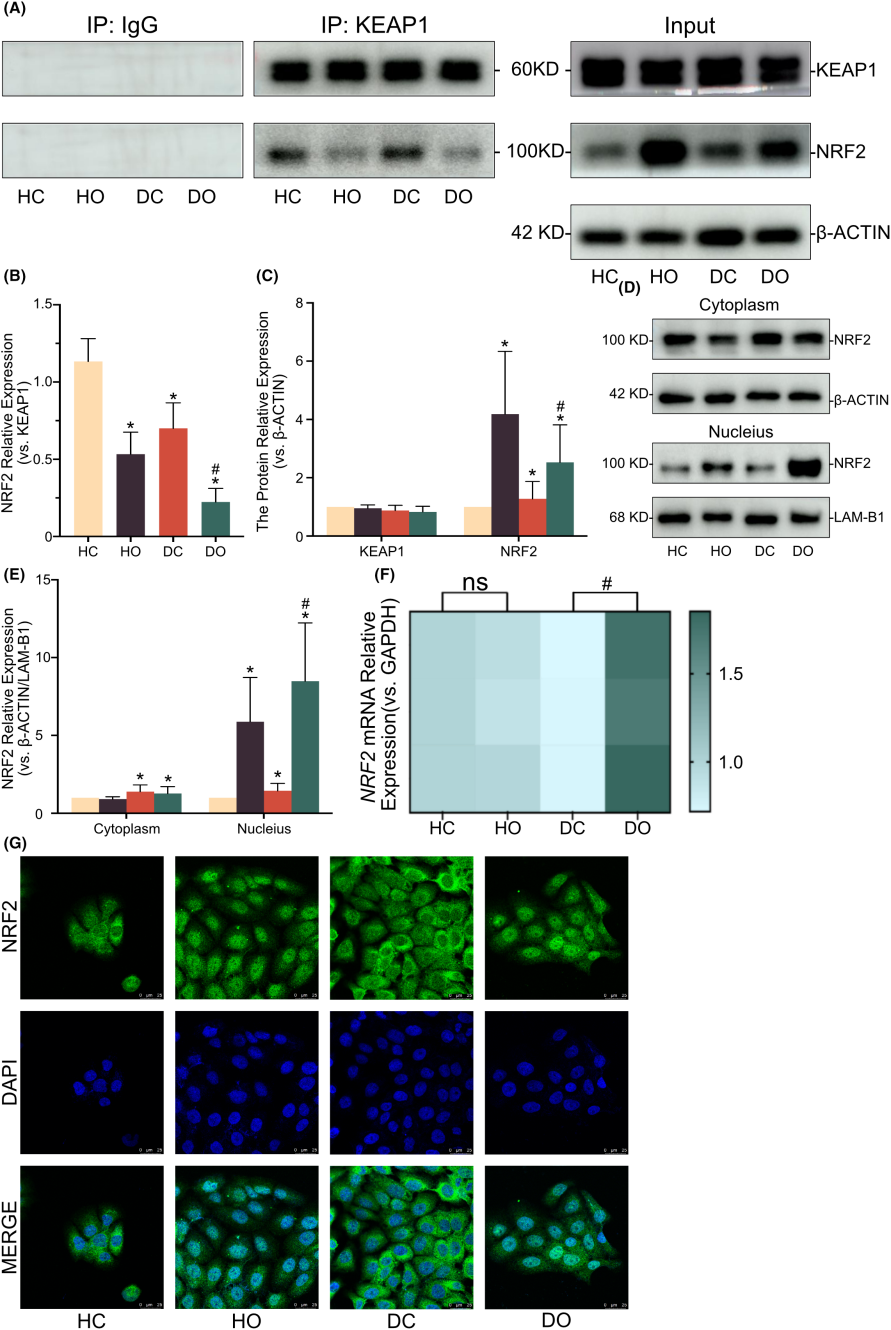
4‐octyl itaconate (OI) activates NRF2 and promotes its nuclear transfer in HaCaT and D66H cells. The combination and separation of KEAP1‐NRF2 revealed by the CO‐IP analysis (A, B). Representative immunoblot analysis showing the expression levels of KEAP1 and NRF2 in the HaCaT and D66H cells (A, C). Representative immunoblot analysis showing expression levels of KEAP1 and NRF2 in the cytoplasm and nucleus, respectively (D, E). The mRNA quantification of *NRF2* with glyceraldehyde‐3‐phosphate dehydrogenase (*GAPDH*) as the loading control (F). The Immunofluorescence showing the expression level and intracellular location of NRF2 (G). All experiments are based on HaCaT control group (HC), HaCaT OI treatment (30 μM and 24 h) group (HO), D66H control group (DC) and D66H OI treatment (30 μM and 24 h) group (DO). The significant difference is determined based on *p* < 0.05 (*) compared with HC and *p* < 0.05 (#) compared with DC. ‘ns’ indicates not significantly different based on *p* > 0.05 compared with HC.

The Co‐IP analysis was performed to verify the alternation of KEAP1‐NRF2 interaction by OI treatment (Figure 4A,B). The whole input protein was detected by Western blot, showing that OI could stimulate the stable expression of protein NRF2 in HaCaT cells without affecting the expression of KEAP1 (Figure 4A,C). Following the OI administration, no discernible change of NRF2 expression was detected in cytoplasm, while a considerable increase in NRF2 expression was observed in the nucleus (Figure 4D,E). The immunofluorescence also revealed that NRF2 protein moved into the nucleus after the OI stimulation (Figure 4G). Furthermore, the results of qPCR analysis revealed no discernible difference in mRNA levels of *NRF2* between the control and the OI‐treated samples (Figure 4F).

**FIGURE 5:**
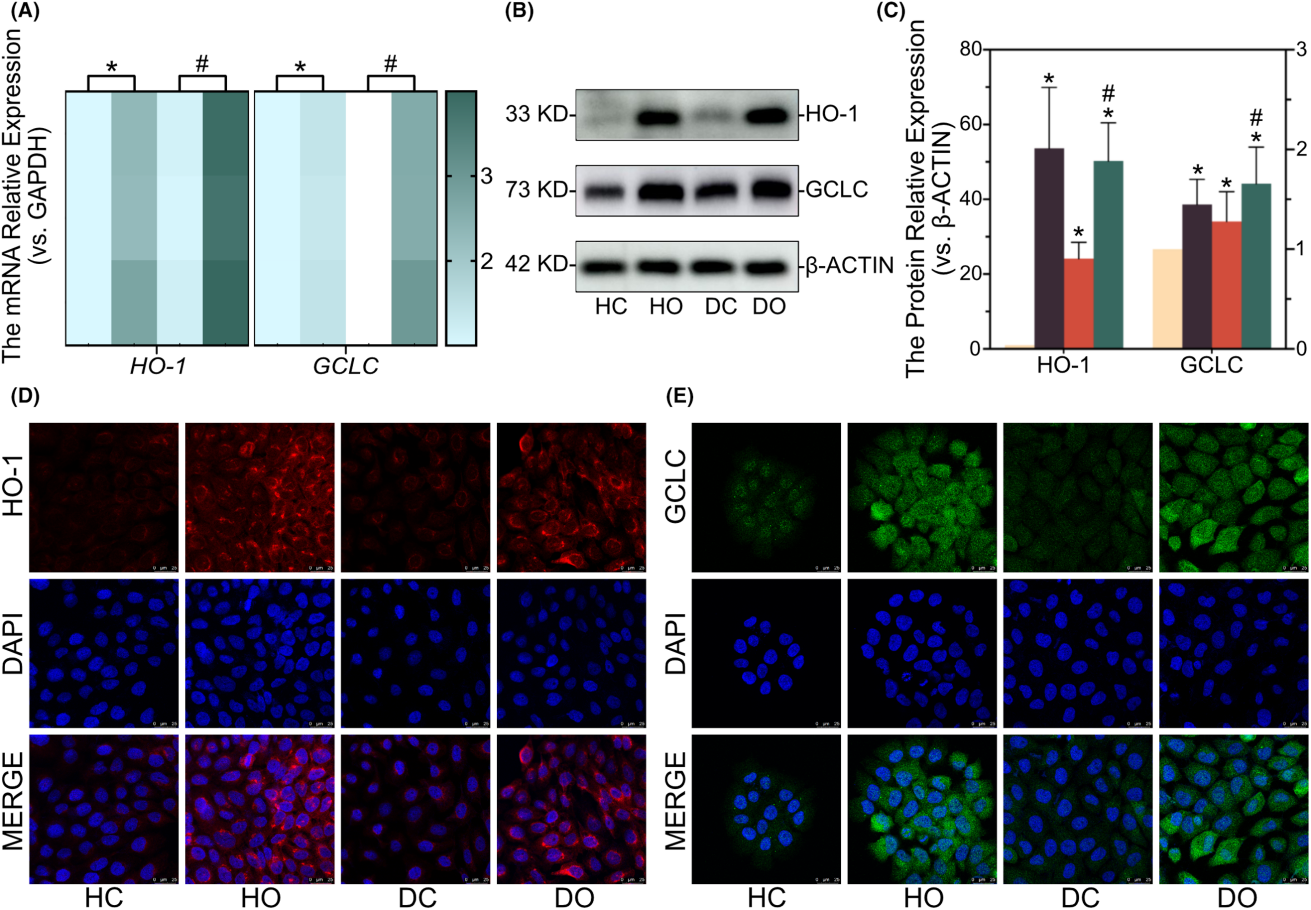
4‐octyl itaconate (OI) promotes the expression of NRF2 downstream targeting protein GCLC/HO‐1 in HaCaT and D66H cells. The mRNA quantification of *HO‐1/GCLC* with *GAPDH* as the loading control (A). Representative immunoblot analysis showing expression levels of HO‐1 and GCLC in the HaCaT and D66H cells (B, C). The Immunofluorescence showing the expression levels of HO‐1 and GCLC (D, E). All experiments are based on HaCaT control group (HC), HaCaT OI treatment (30 μM and 24 h) group (HO), D66H control group (DC), and D66H OI treatment (30 μM and 24 h) group (DO). The significant difference is determined based on *p* < 0.05 (*) compared with HC and *p* < 0.05 (#) compared with DC.

The impact of OI on the two classical NRF2 downstream targets (i.e. *HO‐1* and *GCLC*) was further evaluated, showing increased mRNA levels of both *HO‐1* and *GCLC* after the OI treatment (Figure 5A). Western blot and immunofluorescence results showed that the OI significantly enhanced HO‐1 and GCLC protein expression (Figure 5B–E). These findings implied that OI functioned probably by regulating the KEAP1‐NRF2‐HO1/GCLC signalling pathway in both HaCaT and D66H cells.

### 3.5 Knockdown of NRF2 attenuates the protective effect of OI on cell proliferation arrest, apoptosis and oxidative damage caused by *GJB2* gene mutation

**FIGURE 6:**
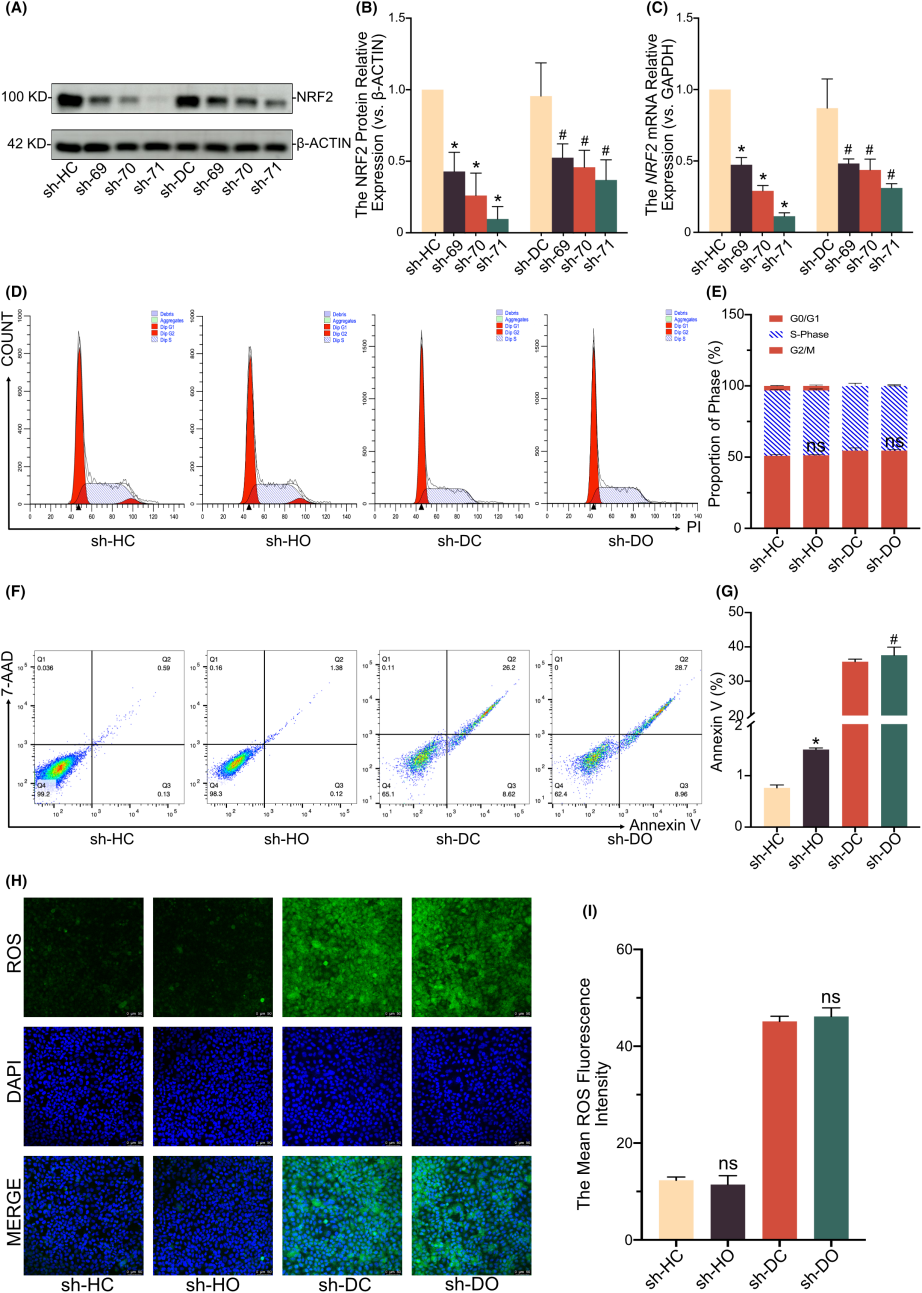
Knockdown of NRF2 attenuates the protective effect of 4‐octyl itaconate (OI) on cell proliferation arrest, apoptosis and oxidative damage caused by *GJB2* mutation. Representative immunoblot analysis showing expression levels of NRF2 after the sh‐RNA transfection in HaCaT and D66H cells (A, B). The mRNA quantification of *NRF2* after sh‐RNA transfection with *GAPDH* as the loading control (C). The FACS analysis of cell cycle and proportion of each phase of the cell cycle (D, E). The FACS analysis of apoptosis and the ratio of Annexin V (F, G). The reactive oxygen species (ROS) staining and fluorescence intensity analysis (H, I). All experiments are based on HaCaT control group with sh‐RNA transfection (sh‐HC), HaCaT OI treatment with sh‐RNA transfection (30 μM and 24 h) group (sh‐HO), D66H control group with sh‐RNA transfection (sh‐DC), and D66H OI treatment with sh‐RNA transfection (30 μM and 24 h) group (sh‐DO). The significant difference is determined based on *p* < 0.05 (*) compared with sh‐HC and *p* < 0.05 (#) compared with sh‐DC. ‘ns’ indicates not significantly different based on *p* > 0.05 compared with sh‐HC or sh‐DC.

The sh‐RNA was transfected into both wild‐type and D66H‐type cells to further verify the above findings. The NRF2 (−/−) was further evaluated based on sh‐71 due to its promising results derived from the Western blotting and qPCR analyses (Figure 6A–C). Blocking NRF2 prevented it from working with or without the presence of OI. Most D66H mutant cells were detected in the G1 phase (Figure 6D,E), whereas the protection of OI from apoptosis and oxidative damage caused by the D66H‐type mutation disappeared (Figure 6F–I). These results confirmed that the NRF2 signalling pathway was indispensable for the protective effect of OI on *GJB2* mutation‐induced cell necrosis, apoptosis and oxidative damage.

## 4 DISCUSSION

It has been reported that the *GJB2* gene mutation (e.g. D66H, c.196G>C) could cause VS in many regions of the skin.^1^, ^9^, ^30^ However, the explicit molecular mechanisms regulating the formation of the skin disease and their potential therapeutic treatments are still rarely reported, especially in the typical palmar keratoderma skin disease. Our study is the natural extension based on our previous study to explore the possible relationship between the *GJB2* gene mutation (i.e. D66H, c.196G>C) and palmar keratoderma skin disease, and the potential treatments of this skin disease.^31^

Numerous studies have shown that the intercellular communication has a major impact on intracellular and extracellular signal transduction, ultimately determining the cell fate. This intercellular communication mainly relies on the junction protein, that is the CX26, which regulates the balance between apoptosis and proliferation to determine the survival of early cells.^32^, ^33^ This process would be possibly explained by oxidation, which was revealed in the hair cell damage caused by *GJB2* gene mutations.^34^ These results were consistent with the findings revealed in our study, showing that the D66H mutation could promote oxidative damage that disturbed the balance between apoptosis and proliferation in keratinized epithelial cells.

As a type of small‐molecule drug, the OI has been shown to play an extensive antioxidant role. In our study, the possible influence of OI on the effects of D66H mutation on cell proliferation arrest, apoptosis and oxidative damage was explored. The results showed that OI could significantly ameliorate the D66H‐type mutation‐induced cell proliferation arrest, apoptosis and oxidative damage, indicating the protective role of OI against D66H mutation. These results showed OI had the same protective effect in other disease models, for example OI protected human umbilical vein endothelial cells from oxidative damage induced by high glucose, protected the cochlea from noise damage, protected the liver from acute liver failure caused by inflammation, oxidative stress, and cell apoptosis and protected the abdominal aorta from the formation of abdominal aortic aneurysm caused by inflammation.^35^, ^36^, ^37^, ^38^

As an effective NRF2 activator, the OI disrupts the stable combination of KEAP1‐NRF2 to release the NRF2 into cells. Then, the NRF2 is translocated to the nucleus to regulate the transcription and expression of related elements, for example GCLC and HO‐1, to perform its antioxidative functions.^20^, ^39^ Our experiments showed that OI treatment induced nuclear transfer of NRF2 and the expression of downstream target genes *GCLC* and *HO‐1* in HaCaT cells. Based on these results, it was hypothesized that the protective effect of OI against D66H mutation‐induced cellular damage was achieved by regulating the KEAP1‐NRF2‐GCLC/HO‐1 pathway. The evidence supporting this hypothesis was collected by knocking down the NRF2. The results showed that after the NRF2 knockdown, the protective effect of OI was significantly attenuated, showing the evident cell proliferation arrest, increased apoptosis and significant ROS production. These results indicated that activation of the KEAP1‐NRF2‐GCLC/HO‐1 signalling pathway was essential for OI rescue of HaCaT cells from damage induced by the D66H‐type mutation.

These results indicated the promising therapeutic prospects of OI for the treatment of skin diseases caused by *GJB2* mutations. Moreover, the activation of the NRF2 signalling pathway could be a potential approach to protecting skin cells from damage. Previous studies have revealed that OI is a fat‐soluble small‐molecule chemical that can penetrate cells without transporters, and keratinocytes are positioned on the skin's surface and may directly touch foreign medications.^24^ This gives OI the possibility to apply now to skin lesions to achieve therapeutic function, avoiding the discomfort produced by intravenous and subcutaneous injection as well as other negative effects.

**FIGURE 7:**
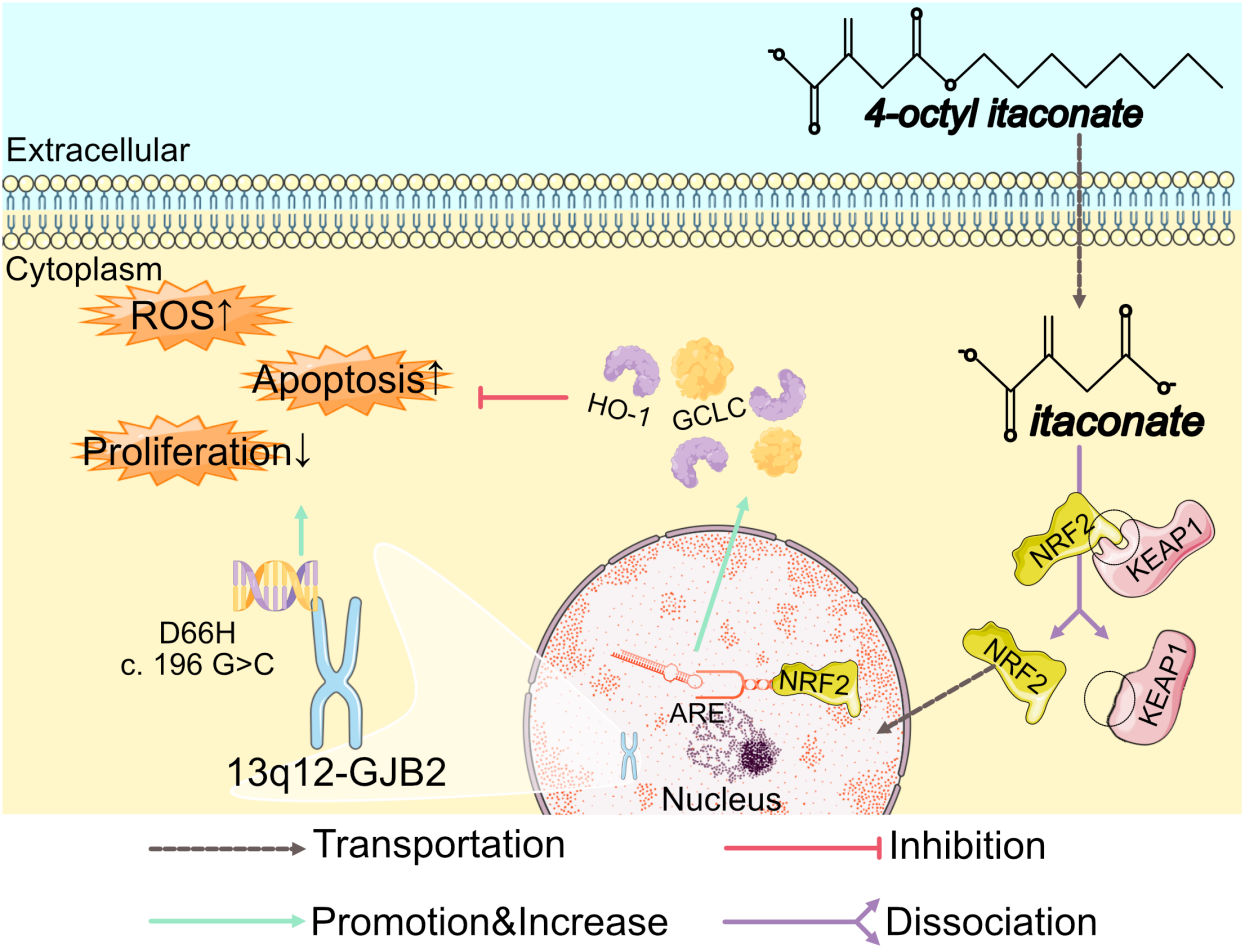
Graphical abstract of the protective effect of 4‐octyl itaconate (OI) on *GJB2* mutation‐induced apoptosis and oxidative damage in HaCaT cells.

In conclusion, this work clearly demonstrated for the first time the possible protective roles of OI and the underlying molecular mechanism in the *GJB2* mutation (i.e. D66H, c.196G>C) induced VS in vitro. After entering into the cells, the OI was converted into itaconate to activate the KEAP1‐NRF2‐GCLC/HO‐1 signalling pathway, inhibiting the oxidative stress that regulated the balance between apoptosis and cell cycle arrest, ultimately protecting the HaCaT cells from damage caused by D66H‐type mutations (Figure 7). 4‐octyl itaconate has promising medical applications for treating conditions related to skin cell damage.

## AUTHOR CONTRIBUTIONS

**Yanrui Chen:** Data curation (lead); methodology (lead); writing – original draft (lead). **Zhenying Wang:** Data curation (supporting); supervision (supporting); writing – original draft (supporting). **Yali Song:** Writing – review and editing (lead). **Nan Chen:** Resources (lead). **Jing Guo:** Formal analysis (lead). **Wenmin Liu:** Writing – review and editing (supporting). **Keying Guo:** Methodology (supporting). **Xia Ling:** Formal analysis (supporting); methodology (supporting). **Li Zhang:** Conceptualization (lead); project administration (lead); supervision (lead); validation (lead).

## CONFLICT OF INTEREST STATEMENT

The authors declare that there are no conflicts of interests.

## Data Availability

The data sets used and/or analyzed in this study can be obtained from the corresponding author on reasonable requirements.

## References

[1] Zhang L , Wang Z , Song Y , Qiu Y , Chen N , Wang Z . D66H mutation in GJB2 gene in a Chinese family with classical Vohwinkel syndrome. Indian J Dermatol Venereol Leprol. 2012;78:640. 22960825 10.4103/0378-6323.100595

[2] GPH L , PCM K , Steijlen PM . The hereditary palmoplantar keratoses: an updated review and classification. Br J Dermatol. 1994;131:1‐14. 8043399 10.1111/j.1365-2133.1994.tb08450.x

[3] Albuloushi A , Lovgren M , Steel A , et al. A heterozygous mutation in GJB2 (Cx26F142L) associated with deafness and recurrent skin rashes results in connexin assembly deficiencies. Exp Dermatol. 2020;29:970‐979. 32866991 10.1111/exd.14187

[4] Korge BP , Ishida‐Yamamoto A , Pünter C , et al. Loricrin mutation in Vohwinkel's keratoderma is unique to the variant with ichthyosis. J Invest Dermatol. 1997;109:604‐610. 9326398 10.1111/1523-1747.ep12337534

[5] Camisa C , Rossana C . Variant of keratoderma hereditaria mutilans (Vohwinkel's syndrome). Treatment with orally administered isotretinoin. Arch Dermatol. 1984;120:1323‐1328. 6237617

[6] Schmuth M , Fluhr JW , Crumrine DC , et al. Structural and functional consequences of loricrin mutations in human loricrin keratoderma (Vohwinkel syndrome with ichthyosis). J Invest Dermatol. 2004;122:909‐922. 15102081 10.1111/j.0022-202X.2004.22431.x

[7] Bondeson M , Nyström A , Gunnarsson U , Vahlquist A . Connexin 26 (GJB2) mutations in two Swedish patients with atypical Vohwinkel (mutilating keratoderma plus deafness) and KID syndrome both extensively treated with acitretin. Acta Derm Venereol. 2006;86:503‐508. 17106596 10.2340/00015555-0164

[8] Iossa S , Chinetti V , Auletta G , et al. New evidence for the correlation of the p.G130V mutation in the GJB2 gene and syndromic hearing loss with palmoplantar keratoderma. Am J Med Genet. 2009;149A:685‐688. 18688874 10.1002/ajmg.a.32462

[9] Bakirtzis G . Targeted epidermal expression of mutant connexin 26(D66H) mimics true Vohwinkel syndrome and provides a model for the pathogenesis of dominant connexin disorders. Hum Mol Genet. 2003;12:1737‐1744. 12837696 10.1093/hmg/ddg183

[10] Gedicke MM , Traupe H , Fischer B , Tinschert S , Hennies HC . Towards characterization of palmoplantar keratoderma caused by gain‐of‐function mutation in loricrin: analysis of a family and review of the literature. Br J Dermatol. 2006;154:167‐171. 16403113 10.1111/j.1365-2133.2005.06995.x

[11] Itin PH , Fistarol SK . Palmoplantar Keratodermas. Clin Dermatol. 2005;23:15‐22. 15708285 10.1016/j.clindermatol.2004.09.005

[12] de Zwart‐Storm EA , van Geel M , Veysey E , et al. A novel missense mutation in GJB2, p.Tyr65His, causes severe Vohwinkel syndrome. Br J Dermatol. 2011;164:197‐199. 20854437 10.1111/j.1365-2133.2010.10058.x

[13] Maestrini E . A missense mutation in connexin26, D66H, causes mutilating keratoderma with sensorineural deafness (Vohwinkel's syndrome) in three unrelated families. Hum Mol Genet. 1999;8:1237‐1243. 10369869 10.1093/hmg/8.7.1237

[14] Snoeckx RL , Hassan DM , Kamal NM , van den Bogaert K , van Camp G . Mutation analysis of the GJB2 (connexin 26) gene in Egypt. Hum Mutat. 2005;26:60‐61. 10.1002/humu.9350 15954104

[15] Alexandrino F , Sartorato EL , Marques‐de‐Faria AP , Steiner CE . G59S mutation in theGJB2 (connexin 26) gene in a patient with Bart‐Pumphrey syndrome. Am J Med Genet. 2005;136A:282‐284. 10.1002/ajmg.a.30822 15952212

[16] Fetoni AR , Zorzi V , Paciello F , et al. Cx26 partial loss causes accelerated presbycusis by redox imbalance and dysregulation of Nfr2 pathway. Redox Biol. 2018;19:301‐317. 30199819 10.1016/j.redox.2018.08.002 PMC6129666

[17] Tocmo R , Parkin K . S‐1‐propenylmercaptocysteine protects murine hepatocytes against oxidative stress via persulfidation of Keap1 and activation of Nrf2. Free Radic Biol Med. 2019;143:164‐175. 31349040 10.1016/j.freeradbiomed.2019.07.022

[18] Wang R , Luo Y , Lu Y , et al. Maggot extracts alleviate inflammation and oxidative stress in acute experimental colitis via the activation of Nrf2. Oxidative Med Cell Longev. 2019;2019:1‐18. 10.1155/2019/4703253 PMC6885191 31827675

[19] Shao Y , Yu H , Yang Y , Li M , Hang L , Xu X . A solid dispersion of quercetin shows enhanced Nrf2 activation and protective effects against oxidative injury in a mouse model of dry age‐related macular degeneration. Oxidative Med Cell Longev. 2019;2019:1‐12. 10.1155/2019/1479571 PMC6875405 31781321

[20] Mills EL , Ryan DG , Prag HA , et al. Itaconate is an anti‐inflammatory metabolite that activates Nrf2 via alkylation of KEAP1. Nature. 2018;556:113‐117. 29590092 10.1038/nature25986 PMC6047741

[21] Bambouskova M , Gorvel L , Lampropoulou V , et al. Electrophilic properties of itaconate and derivatives regulate the IκBζ‐ATF3 inflammatory axis. Nature. 2018;556:501‐504. 29670287 10.1038/s41586-018-0052-z PMC6037913

[22] Uyguner O , Tukel T , Baykal C , et al. The novel R75Q mutation in theGJB2gene causes autosomal dominant hearing loss and palmoplantar keratoderma in a Turkish family. Clin Genet. 2002;62:306‐309. 12372058 10.1034/j.1399-0004.2002.620409.x

[23] Xu K , Chen S , Xie L , et al. The protective effects of systemic dexamethasone on sensory epithelial damage and hearing loss in targeted Cx26‐null mice. Cell Death Dis. 2022;13:545. 35688810 10.1038/s41419-022-04987-3 PMC9187686

[24] Li R , Zhang P , Wang Y , Tao K . Itaconate: a metabolite regulates inflammation response and oxidative stress. Oxidative Med Cell Longev. 2020;2020:1‐11. 10.1155/2020/5404780 PMC7382747 32724492

[25] Liu H , Feng Y , Xu M , Yang J , Wang Z , Di G . Four‐octyl itaconate activates Keap1‐Nrf2 signaling to protect neuronal cells from hydrogen peroxide. Cell Commun Signal. 2018;16:81. 30442144 10.1186/s12964-018-0294-2 PMC6238317

[26] Zhang Q , Bai X , Wang R , et al. 4‐octyl itaconate inhibits lipopolysaccharide (LPS)‐induced osteoarthritis via activating Nrf2 signalling pathway. J Cell Mol Med. 2022;26:1515‐1529. 35068055 10.1111/jcmm.17185 PMC8899168

[27] Xie Y , Chen Z , Wu Z . Four‐octyl itaconate attenuates UVB‐induced melanocytes and keratinocytes apoptosis by Nrf2 activation‐dependent ROS inhibition. Oxidative Med Cell Longev. 2022;2022:1‐13. 10.1155/2022/9897442 PMC8933077 35308171

[28] Michaličková D , Hrnčíř T , Canová NK , Slanař O . Targeting Keap1/Nrf2/ARE signaling pathway in multiple sclerosis. Eur J Pharmacol. 2020;873:172973. 32017935 10.1016/j.ejphar.2020.172973

[29] Yi Z , Deng M , Scott MJ , et al. Immune‐responsive gene 1/itaconate activates nuclear factor erythroid 2‐related factor 2 in hepatocytes to protect against liver ischemia‐reperfusion injury. Hepatology. 2020;72:1394‐1411. 31997373 10.1002/hep.31147 PMC7702080

[30] Castro PJS , Fernandez CN , Subirana PQ , Ortiz MP . Vohwinkel syndrome secondary to missense mutation D66H in GJB2 gene (connexin 26) can include epileptic manifestations. Seizure. 2010;19:129‐131. 20031451 10.1016/j.seizure.2009.11.009

[31] Lu Y , Zhang R , Wang Z , et al. Mechanistic effect of the human GJB6 gene and its mutations in HaCaT cell proliferation and apoptosis. Braz J Med Biol Res. 2018;51:e7560. 30043857 10.1590/1414-431X20187560 PMC6065815

[32] Decrock E , Vinken M , de Vuyst E , et al. Connexin‐related signaling in cell death: to live or let die? Cell Death Differ. 2009;16:524‐536. 19197295 10.1038/cdd.2008.196

[33] Gilleron J , Carette D , Segretain D , Pointis G . Multiple and complex influences of connexins and pannexins on cell death. Biochim Biophys Acta Biomembr. 2018;1860:182‐191. 28625689 10.1016/j.bbamem.2017.06.004

[34] Yu X , Li S , Ding Y . Maternally transmitted nonsyndromic hearing impairment may be associated with mitochondrial tRNA^Ala^ 5601C>T and tRNA^Leu(CUN)^ 12311T>C mutations. J Clin Lab Anal. 2022;36:e24298. 35218233 10.1002/jcla.24298 PMC8993639

[35] Tang C , Tan S , Zhang Y , Dong L , Xu Y . Activation of Keap1‐Nrf2 signaling by 4‐octyl itaconate protects human umbilical vein endothelial cells from high glucose. Biochem Biophys Res Commun. 2019;508:921‐927. 30545629 10.1016/j.bbrc.2018.12.032

[36] Fetoni AR , Paciello F , Rolesi R , et al. Rosmarinic acid up‐regulates the noise‐activated Nrf2/HO‐1 pathway and protects against noise‐induced injury in rat cochlea. Free Radic Biol Med. 2015;85:269‐281. 25936352 10.1016/j.freeradbiomed.2015.04.021

[37] Li R , Yang W , Yin Y , Zhang P , Wang Y , Tao K . Protective role of 4‐octyl itaconate in murine LPS/D‐GalN‐induced acute liver failure via inhibiting inflammation, oxidative stress, and apoptosis. Oxidative Med Cell Longev. 2021;2021:1‐11. 10.1155/2021/9932099 PMC8387163 34457120

[38] Song H , Xu T , Feng X , et al. Itaconate prevents abdominal aortic aneurysm formation through inhibiting inflammation via activation of Nrf2. EBioMedicine. 2020;57:102832. 32574955 10.1016/j.ebiom.2020.102832 PMC7322255

[39] Peace CG , O'Neill LAJ . The role of itaconate in host defense and inflammation. J Clin Invest. 2022;132:e148548. 35040439 10.1172/JCI148548 PMC8759771

